# Removal of proximally migrated pancreatic stents: Ten-year experience from a large endoscopy center and literature review

**DOI:** 10.1055/a-2870-7506

**Published:** 2026-06-01

**Authors:** Zhezhe Fan, Fanyang Kong, Fan Yang, Hongyuan Zou, Zhengdong Li, Yang-Yang Qian, Feng Li, Lei Wang

**Affiliations:** 1 College of Basic Medical Sciences 12521 Naval Medical UniversityShanghaiShanghaiChina; 2 Department of Gastroenterology 12520 Changhai HospitalShanghaiShanghaiChina; 3 Department of Gastroenterology and HepatologyChristus Trinity Mother Frances Hospital and ClinicTexasUnited States

**Keywords:** Pancreatobiliary (ERCP/PTCD), ERC topics, Strictures, Quality and logistical aspects, Image and data processing, documentatiton

## Abstract

**Background and study aims:**

With widespread application of pancreatic stent in managing pancreatic diseases and preventing post-endoscopic retrograde cholangiopancreatography (ERCP) pancreatitis, incidence of stent-related complications such as proximal migration has substantially increased. This study aimed to provide strategic insights for retrieving proximally migrated pancreatic stents by analyzing a large endoscopy center experience and synthesizing novel techniques.

**Patients and methods:**

A retrospective analysis was conducted over 10 years, including 43 cases of proximally migrated pancreatic stent among 32,537 ERCP procedures (2014–2024). Patient demographics, stent features, and procedure details were assessed. Literature on advanced retrieval techniques was reviewed.

**Results:**

Most patients (67.4%) were asymptomatic; epigastric discomfort occurred in 32.6%. Endoscopic retrieval was successful in 81.4% of cases (35/43), with 58.1% (25/43) achieved using a single accessory. Median operation duration was 35 minutes (interquartile range 24–59). Pancreatic duct pre-dilation significantly shortened the procedure (31 ± 9 vs. 47 ± 13 minutes, *P* < 0.001). Stent migration duration showed no correlation with procedure difficulty (r = 0.131, *P* = 0.201). Eight cases (18.6%) met criteria for difficult-to-retrieve stents (DTRSs), mainly due to ductal tortuosity, strictures, or migration into branch pancreatic ducts. All procedures were completed without perioperative complications.

**Conclusions:**

Endoscopic retrieval is safe and effective for proximal pancreatic stent migration. Success optimization requires careful preoperative evaluation, ductal preparation, and adoption of advanced techniques for difficult cases. The DTRS concept provides a valuable framework for clinical decision-making when standard retrieval fails.

## Introduction

Pancreatic stents, typically deployed via endoscopic retrograde cholangiopancreatography (ERCP) or endoscopic ultrasound (EUS) 1 , serve as critical therapeutic conduits for pancreatic duct drainage and obstruction relief. These devices play a pivotal role in multiple scenarios, including chronic pancreatitis (CP) pain management, post-ERCP pancreatitis prophylaxis 2 , and treatment of pancreatic duct obstruction arising from stones or neoplastic processes, as well as in managing pancreatic pseudocysts and pancreas divisum. Although their utility is well-established, stent-related complications persist as a notable clinical challenge 3 4 , with stent migration emerging as one of the most prevalent adverse events (AEs) encountered in daily practice.

Pancreatic stent migration presents in two distinct patterns with important therapeutic implications. Distal migration (incidence: ~7.5%), characterized by stent migration into the intestinal lumen, typically follows a benign course with spontaneous passage in most cases 5 . In contrast, proximal migration (incidence: ~4.7%), involving retrograde movement toward the pancreatic tail 6 , represents a more significant event that often requires endoscopic or surgical intervention due to potential complications including ductal injury, recurrent pancreatitis, or abscess formation. This dichotomy in clinical behavior underscores the need for differential management strategies based on migration directionality.

A prerequisite for suitability of endoscopic measures is presence of a dilated pancreatic duct system 7 . However, endoscopic retrieval of proximally migrated stents poses substantial technical challenges due to anatomical constraints, characterized by strictures and tortuosities 8 . Furthermore, migrated stents may undergo structural compromise, manifesting as deformation, fragility, or fracture, which exacerbates retrieval difficulty 9 . Therefore, retrieval of proximally migrated stents represents a critical priority.

This study synthesized institutional experience from a large endoscopy center specializing in pancreatic disorders over the past decade and reviews advances in retrieval techniques reported over the past 5 years. By integrating retrospective case analysis with recent evidence, we aimed to establish a strategic framework for managing proximally migrated pancreatic stents, addressing both technical and clinical difficulties.

## Patients and methods

### Study design and patient selection

This single-center retrospective cohort study evaluated patients with proximally migrated pancreatic stent managed at the Endoscopy Center of Changhai Hospital between January 2014 and January 2024. Inclusion criteria included: 1) radiologically and endoscopically confirmed pancreatic stent migration; 2) undergoing endoscopic retrieval of stent; and 3) complete data on information of patient, stent and operation. Exclusion criteria included confirmed distal stent migration (intestinal migration) and loss to follow-up. A total of 32,537 patients who underwent ERCP during the 10-year study period were initially identified. After applying the inclusion and exclusion criteria, 43 patients with proximally migrated pancreatic stents undergoing endoscopic retrieval were included in the final analysis.

### Data collection and processing

Study data were systematically extracted from the electronic medical records and categorized into three domains: 1) patient characteristics, comprising demographic variables (sex, age), clinical parameters (post-endoscopic sphincterotomy [EST] status, primary diagnosis), symptomatology (perioperative and post-migration symptoms), anatomical variations (pancreatic duct anomalies), and baseline data of retrieval-failure cases; 2) stent characteristics, including migration duration, type (single-pigtail stent, straight stents with internal and external flaps, straight stents with external flaps, straight-type stent), dimensions (length, diameter), stent anomalies, and positioning of the stent with quantitative measurements derived from digital X-ray archives using institutional software; and 3) procedure characteristics, encompassing operation duration, stent retrieval success rate, instrumentation details, and ductal pre-dilation status. In this study, baseline data and operational-related information for the 43 patients with proximal pancreatic stents included in the analysis were statistically evaluated and compared. Based on the new age categorization proposed by the World Health Organization, patients were divided into three groups: ≤ 44 years, 45 to 59 years, and ≥ 60 years. To evaluate difficulty of stent retrieval, we quantified it using operation duration. Conventional endoscopic retrieval techniques used in this study included traction-based methods (forceps, baskets, and snares) and friction-based methods (balloon catheters), which were selected according to endoscopist preference and procedural conditions.

### Definition of “difficult-to-retrieve stent”

We introduced the term “difficult-to-retrieve stent” to characterize cases where pancreatic stent removal fails despite ≥ 3 standard retrieval attempts. An “attempt” was operationally defined as the complete cycle of: 1) successful deep cannulation of the pancreatic duct; 2) advancement of a retrieval device (e.g., balloon, forceps, basket) to engage the stent under fluoroscopic guidance; and 3) application of sustained traction. A change of the retrieval device or a significant alteration in technique (e.g., switching from traction-based to friction-based retrieval) constituted a new attempt. This operational definition serves three key purposes: 1) establishing an objective threshold for procedural difficulty; 2) enabling early identification of cases requiring advanced techniques; and 3) informing risk-adapted procedural strategies to minimize complications.

### Patient follow-up

All patients were enrolled in follow-up, with eight cases of unsuccessful stent retrieval undergoing longitudinal surveillance (6–24 months post-procedure). Follow-up assessments included: 1) symptom evaluation (epigastric discomfort); 2) assessment of retained stent-related complications (obstruction, infection, pancreatic duct injury); and 3) quality-of-life metrics (activity restrictions). Follow-up data were collected through outpatient clinic visits and telephone interviews. Primary study outcomes were occurrence of AEs associated with retained stents and subsequent therapeutic interventions.

### Search strategy

To assess technical advancements in retrieval of proximally migrated stent over the past 5 years (January 2020–December 2024), a systematic literature search was conducted using PubMed. During the search, the terms with wildcard “*” were used: pancreas*, stent*, displace*, migrate*, shift*, retrieve* and remove*. The following search string was: ((pancreas[All Fields]) OR (pancreases[All Fields]) OR (pancreatic[All Fields])) AND ((stent[All Fields]) OR (stents[All Fields])) AND ((displace[All Fields]) OR (displaced[All Fields]) OR (displacement[All Fields]) OR (displacements[All Fields]) OR (migrate[All Fields]) OR (migrated[All Fields]) OR (migration[All Fields]) OR (migrations[All Fields]) OR (shift[All Fields]) OR (shifted[All Fields]) OR (shifts [All Fields])) AND ((retrieval[All Fields] OR retrieve[All Fields] OR removal[All Fields] OR remove[All Fields])).

**Fig. 1:**
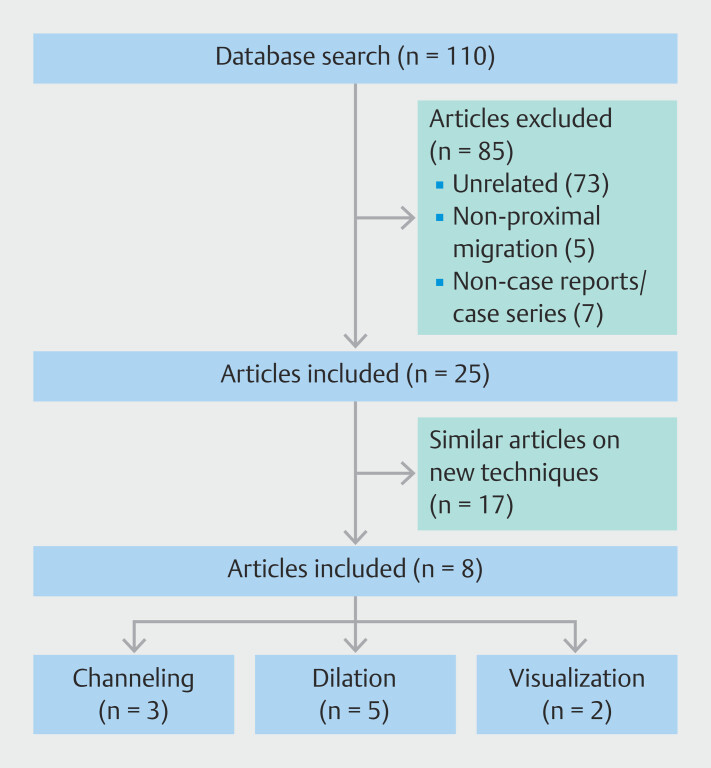
Literature search, review and analysis.

After screening 110 records, unrelated studies, non-proximal migration, or non-case reports/case series were excluded. The selected studies focused on novel techniques for the retrieval of proximally migrated stents, and the relevant search process was shown ( Fig. 1 ). Eight studies were included and their retrieval techniques were summarized.

### Statistical analysis

Data analysis was performed using IBM SPSS Statistics 25.0 (IBM Corp.). Continuous variables were assessed for normality via the Shapiro-Wilk test and reported as mean ± standard deviation or median (interquartile range, [IQR]) for parametric and non-parametric distributions (e.g., migration duration, operation duration), respectively. Categorical variables were expressed as frequencies and percentages. Between-group comparisons (e.g., ductal pre-dilation vs. non-ductal pre-dilation) were analyzed using independent t-tests for normally distributed data. Associations between operation duration and stent migration duration were evaluated using Pearson’s correlation coefficient (r) with a two-tailed significance threshold of *P* < 0.05.

## Results

### Patient characteristics

A retrospective analysis of 32,537 ERCP procedures performed at our center over a 10-year period revealed 43 cases involving retrieval of proximally migrated pancreatic stents. Among them, there were males (62.8%, 27/43) and females (37.2%, 16/43). Median age of patients was 48 years (range 7–77) and the majority of patients being younger than age 60 years (88.4%, 38/43). The primary disease in most patients was CP (93.0%, 40/43), of which 26 cases (60.5%) were complicated by pancreatic stones. Other diagnoses comprised pancreatic space-occupying lesions (4.7%, 2/43) and annular pancreas (2.3%, 1/43). EST was performed in 90.7% of patients (39/43) with migrated pancreatic stents. Most cases (67.4%, 29/43) were asymptomatic, whereas 32.6% (14/43) presented with upper abdominal discomfort. Pancreatic duct abnormalities were detected in 39 cases (90.7%), consisting of ductal strictures (72.1%, 31/43) and tortuosity (18.6%, 8/43). Pancreatic duct tortuosity exhibited irregular morphologies, including α-shaped, S-shaped, and acute-angle configurations. The majority of pancreatic duct strictures (28/31, 90.3%) were located in the pancreatic head, whereas a minority (3/31, 9.7%) were found in the pancreatic body ( Table 1 ).

**Table:** **Table 1** Summary of patient characteristics.

**Characteristic**	**n/N (%)**
Sex	
Male	27/43 (62.8%)
Female	16/43 (37.2%)
Age	
≤ 44 years	18/43 (41.9%)
45~59 years	20/43 (46.5%)
≥ 60 years	5/43 (11.6%)
Post-EST	
Yes	39/43 (90.7%)
No	4/43 (9.3%)
Primary diagnosis	
CP with pancreatic stones	26/43 (60.5%)
CP without pancreatic stones	14/43 (32.6%)
Pancreatic space-occupying lesion	2/43 (4.7%)
Annular pancreas	1/43 (2.3%)
Symptoms after stent migration	
Upper abdominal discomfort	14/43 (32.6%)
No discomfort	29/43 (67.4%)
Pancreatic duct anomalies	
Pancreatic duct tortuosity	8/43 (18.6%)
Pancreatic duct strictures	31/43 (72.1%)
Normal	4/43 (9.3%)
Type of pancreatic duct tortuosity	
Irregular tortuosity	4/8 (50.0%)
α-shaped tortuosity	1/8 (12.5%)
S-shaped tortuosity	2/8 (25.0%)
Acute-angle tortuosity	1/8 (12.5%)
Site of pancreatic duct stricture	
Pancreatic head	28/31 (90.3%)
Pancreatic body	3/31 (9.7%)
EST, endoscopic sphincterotomy.	

In our cohort, eight patients exhibited unsuccessful stent retrieval, with the following characteristics: pancreatic duct tortuosity (n = 2), migration into branch pancreatic ducts (n = 3), stent tortuosity (n = 3), deep migration to the pancreatic body (n = 1), and pancreatic duct strictures (n = 1). Among the eight failures, follow-up duration ranged from 6 to 24 months; at ≥ 12 months, three still retained migrated stents, with at least one additional ERCP retrieval attempt performed either at our center or other institutions. Among them, two cases reported intermittent episodes of post-activity abdominal pain or recurrent acute pancreatitis. The remaining five patients subsequently underwent successful ERCP removal of the migrated stents ( Table 2 ). All eight cases met predefined criteria for difficult-to-retrieve stent, having failed retrieval after three or more standardized attempts as defined in Methods.

**Table:** **Table 2** Clinical profiles of unsuccessful stent retrieval cases.

**Case**	**Type of difficulty**	**Migration duration (days)**	**Clinical consequence**	**Stent type**	**Operation duration (min)**	**Salvage management**	**Post-retrieval symptoms**	**Single accessory**	**Multiple accessories**
1	Migration into branch pancreatic duct	195	Asymptomatic	Straight stent with internal and external flaps	76	Guidewire-guided placement of COOK nasopancreatic drainage tube (pancreatic fluid output); no further retrieval.	None	–	×
2	Migration into branch pancreatic duct	108	Asymptomatic	Single-pigtail stent	15	Placement of 5 Fr × 8 cm COOK SPSOF single-pigtail stent via guidewire.	None	×	–
3	Stent tortuosity, pancreatic duct tortuosity (S-shaped), migration into branch pancreatic duct	169	Abdominal pain	Single-pigtail stent	59	Placement of 5 Fr × 5 cm COOK SPSOF stent; no further intervention.	Activity-induced pain (lifting/cycling) at migration site	–	×
4	Stent tortuosity	496	Asymptomatic	Single-pigtail stent	35	Guidewire-assisted placement of 7F × 7 cm COOK SPSOF single-pigtail stent.	None	–	×
5	Stent tortuosity	357	Asymptomatic	Single-pigtail stent	25	No intervention	None	–	×
6	Pancreatic duct tortuosity (α-shaped)	546	Abdominal pain	Single-pigtail stent	51	No intervention	None	–	×
7	Deep migration to the pancreatic body	153	Asymptomatic	Single-pigtail stent	166	Successful retrieval via ERCP after 1 year	Acute exacerbation of CP	–	×
8	Pancreatic duct stricture	41	Asymptomatic	Straight stent with internal and external flaps	53	Guidewire-assisted placement of 7F × 8 cm COOK single-pigtail stent.	None	–	×
ERCP, endoscopic retrograde pancreatography.									

### Stent characteristics

Median migration duration for stents was 291 days (IQR 112–431). The migrated stents comprised primarily single-pigtail pancreatic stents (37.2%, 16/43) and straight stents with internal and external flaps (41.9%, 18/43), with fewer straight stents with external flaps (14.0%, 6/43) and straight-type stents (7.0%, 3/43). Stent diameters were most commonly 5F (41.9%, 18/43) and 8.5 Fr (32.6%, 14/43), whereas predominant lengths were 5 cm (46.5%, 20/43) and 7 cm (39.5%, 17/43). Migration occurred mainly in the main pancreatic duct (MPD) (86.1%, 37/43), with only six cases (14.0%) involving the accessory pancreatic duct. Stent-related complications were identified in 18 patients (41.9%), including stent tortuosity (10/43, 23.3%), migration into branch pancreatic duct (11.6%, 5/43), and mechanical fractures (7.0%, 3/43) confirmed by radiographic evidence of stent discontinuity ( Table 3 ).

**Table:** **Table 3** Summary of stent characteristics.

**Characteristic**	**n/N (%)**
Migration duration, median (IQR), day	291 (112–431)
Type	
Single-pigtail pancreatic stent	16/43 (37.2%)
Straight stents with internal and external flaps	18/43 (41.9%)
Straight stents with external flaps	6/43 (14.0%)
Straight-type pancreatic stent	3/43 (7.0%)
Length	
5 cm	20/43 (46.5%)
7 cm	17/43 (39.5%)
Others*	6/43 (14.0%)
Diameter	
5F	18/43 (41.9%)
7F	6/43 (14.0%)
8.5F	14/43 (32.6%)
10F	5/43 (11.6%)
Stent anomalies	
Stent tortuosity	10/43 (23.3%)
Migration into branch pancreatic duct	5/43 (11.6%)
Stent fracture	3/43 (7.0%)
Normal stent	25/43 (58.1%)
Positioning of the stent	
MPD stent	37/43 (86.1%)
Accessory pancreatic duct stent	6/43 (14.0%)
*Missing data: 4 cm (n = 1); 9 cm (n = 1); 10 cm (n = 1); 12 cm (n = 3). IQR, interquartile range; MPD, main pancreatic duct.	

### Operational outcomes

Median operation duration for endoscopic retrieval of migrated stents in this cohort was 35 minutes (IQR 24–59). Regression analysis demonstrated that there was no correlation between migration duration and operation duration (Pearson’s r = 0.131, *P* = 0.201; Durbin-Watson statistic = 2.002). The overall success rate of endoscopic stent removal was 81.4% (35/43). Among the 43 cases, 25 were single endoscopic accessory. A success rate of 58.1% (25/43) was achieved with use of single endoscopic accessory strategies, including rat-tooth forceps, biopsy forceps, baskets, and balloons. In the remaining 18 of 43 initial failures, 10 of 18 (55.6%) were rescued by using multiple endoscopic accessories (≥ 2 devices). An additional analysis assessed the association between pancreatic duct pre-dilation and operative outcomes. Pancreatic duct pre-dilation significantly reduced operation duration, with mean durations of 31 ± 9 minutes in the dilation group vs. 47 ± 13 minutes in the non-dilation group ( *P* < 0.001). The technical success rate also increased from 76.5% (13/17) in the non-dilated group to 84.6% (22/26) in the dilated group, although this difference was not statistically significant ( *P* > 0.05) ( Table 4 ).

**Table:** **Table 4** Summary of operational characteristics.

**Characteristic**	**n/N (%)**
Operation duration, median (IQR), min	35 (24–59)
Overall	
Successful retrieval	35/43 (81.4%)
ail retrieval	8/43 (18.6%)
Single endoscopic accessory strategy	
Successful cases	25/43 (58.1%)
Multiple endoscopic accessories strategy	
Successful cases	10/18 (55.6%)
Fail cases	8/18 (44.4%)
Ductal pre-dilation	
Successful retrieval	22/26 (84.6%)
Fail retrieval	4/26 (15.4%)
Non-ductal pre-dilation	
Successful retrieval	13/17 (76.5%)
Fail retrieval	4/17 (23.5%)
IQR, interquartile range.	

## Discussion

Previous studies have shown that proximally migrated pancreatic stent arises from four key factors: anatomical issues 10 11 (e.g., sphincterotomy, duct fibrosis, acute angulation); technical elements 12 (inexperience, deep placement, residual stones); stent properties 6 10 11 (excessive length/diameter, straight design, flexibility); and biomechanical forces (juice flow, tumor shrinkage). Although our study did not perform a risk-factor analysis, distribution of stent types and high post-EST prevalence (90.7%) in our cohort is consistent with previously reported patterns. These findings highlight the importance of preventive strategies when these risk factors are present.

Clinical presentation of proximal stent migration is often nonspecific. Although previous reports have associated stent migration with pancreatic duct dilation and elevated intraductal pressure 13 and symptomatic presentations such as acute pancreatitis 14 15 , our finding that 67.4% of patients remained asymptomatic aligns with series describing migration as an incidental imaging finding 16 . Besides, our analysis showed no significant correlation between migration duration and procedure difficulty (r = 0.131, *P* = 0.201). Among the 35 patients with successful stent retrieval, the longest stent migration duration reached 9 years, with operation duration of 16 minutes (median operation duration: 35 minutes). These findings suggest that long-standing migration does not necessarily preclude successful endoscopic removal.

Current management of proximally migrated pancreatic stents primarily involves endoscopic retrieval or surgical intervention. Endoscopic removal, predominantly via ERCP, is favored for its minimally invasive nature and cost-effectiveness. Supplementary techniques such as pancreaticoscopy and EUS-guided retrieval further expand therapeutic options 17 18 . Our overall endoscopic retrieval success rate of 81.4% (35/43) corresponds closely with the 83% success rate reported by other studies 11 . In contrast, surgical management, although definitive, carries greater invasiveness and higher morbidity, including risks of pancreatic fistula and wound infection.

Our findings support a delayed repeat ERCP strategy rather than immediate surgical intervention, particularly given the predominantly asymptomatic nature of retained stents. Previous studies have confirmed retained pancreatic duct foreign bodies, including guidewires, often remain asymptomatic over extended periods 19 . In our cohort, five patients with initial retrieval failure eventually achieved successful removal during repeat ERCP. A representative case (Case 8) underwent repeat ERCP 5 months after the initial attempt. Intraoperative findings revealed distal migration of the stent compared with its position 5 months prior, which we hypothesize may be attributed to pancreatic juice hydrodynamics or increased proximal ductal pressure gradients. The stent was ultimately retrieved successfully using forceps. These observations suggest that repeat ERCP after temporary retention can be a safe and feasible strategy.

In cases of unsuccessful retrieval, placement of an additional pancreatic stent is routinely considered in our practice. This approach serves two purposes. First, repeated and prolonged manipulation within the pancreatic duct during retrieval attempts may increase intraductal pressure and risk of post-ERCP pancreatitis; temporary pancreatic stenting may help reduce this risk by facilitating ductal drainage. Second, a retained migrated stent may partially obstruct the proximal pancreatic duct or the papillary region, particularly if edema develops after the procedure, potentially impairing pancreatic juice outflow. Placement of a temporary pancreatic stent, therefore, may help maintain ductal patency. In general, the stent selected for this purpose should be short and of small caliber to provide adequate drainage while minimizing ductal irritation. During placement, careful fluoroscopic monitoring is essential to avoid inadvertently pushing the retained stent further proximally toward the pancreatic tail, which could increase risk of pancreatic duct injury or perforation.

When cannulation via the major papilla is not feasible due to mechanical obstruction or anatomic anomalies of the MPD, a stent may be placed via the minor papilla into the accessory pancreatic duct. In our study, we observed six cases involving accessory pancreatic duct stents. Among these, three cases exhibited pancreatic duct tortuosity, and one had annular pancreas, characterized by the pancreatic duct encircling the descending duodenum before turning left. Despite the anatomical challenges of the accessory pancreatic duct—such as a narrower orifice and less defined ductal architecture—all migrated stents were successfully retrieved 20 . Conventional retrieval devices, including balloon catheters, were effective without procedure-related complications, indicating that retrieval strategies for accessory pancreatic duct stents are comparable to those for MPD stents.

Conventional endoscopic retrieval relies on traction-based techniques (forceps, baskets, snares) or friction-based techniques (balloon catheters). While previous studies identified biopsy forceps as the first-line approach, utilized in up to 55% of cases 11 , our data show a preference for balloon-assisted retrieval, which succeeded as a single accessory in 58.1% of cases. Balloon catheters provide the advantage of minimal ductal irritation while simultaneously dilating strictured segments. When a single accessory fails, multiple devices are often used in combination, consistent with strategies reported in previous studies 21 . However, 18.6% of stents (8/43) met our criteria for difficult-to-retrieve stent. In the overall cohort, pancreatic duct strictures and tortuosity were common (31/43, 72.1% and 8/43, 18.6%, respectively). Among the eight difficult-to-retrieve stent cases, duct-related factors were also frequently present (tortuosity, n = 2; stricture, n = 1). Strictures and tortuosity frequently impede full opening of instruments, making stent removal challenging and emphasizing the necessity for advanced techniques in such complex situations.

Migration into branch pancreatic duct represents a particular challenge in stent retrieval. In our series, successful retrieval was achieved in only two of five cases, with failures resulting from firmly embedded stents or intraoperative migration into branch pancreatic duct, precluding successful removal. Thus, during retrieval of proximally migrated stents, caution is essential to prevent branch pancreatic duct migration; if it occurs, the stent is gently pushed inward with a balloon to reposition it into the MPD for standard removal. In the cases of stent tortuosity, five were successfully removed using rat-tooth forceps or foreign body forceps to grip the distal end of the stent directly, whereas balloon had limited application in these cases. We have also conducted relevant analyses regarding optimization strategies prior to removal. Ductal pre-dilation emerged as a critical optimization strategy, reducing mean operation duration by 34% ( *P* < 0.001) in stenotic ducts. At our institution, CP patients routinely undergo balloon catheter or bougie dilator pre-dilation to dilate the pancreatic duct prior to pancreatic stent retrieval therapy.

There were a considerable number of patients requiring ERCP-guided pancreatic stone retrieval treated at our center. Pancreatic stones may act as foreign bodies obstructing the retrieval process of proximally migrated pancreatic stents. Based on our clinical experience, the treatment strategy was tailored according to the stone burden. For cases with proximally migrated stent combined with larger or impacted stones, initial extracorporeal shock wave lithotripsy was preferred to fragment the stones, thereby reducing frictional resistance and facilitating subsequent endoscopic stent retrieval. In situations in which stone burden was minimal and not obstructing stent removal, direct endoscopic intervention was often feasible. Subsequent ERCP procedures addressing both stones and migrated stents simultaneously can reduce the number of required endoscopic interventions during hospitalization 22 .

Our literature review reveals that recent innovations in endoscopic retrieval techniques primarily address two procedural challenges: establishing stable channel and dilating stenotic segments. For ductal access, techniques such as biliary stent pusher tube stabilization 23 and novel delivery systems for micro-forceps 24 have demonstrated efficacy in narrow ducts. In managing ductal strictures, methods including cystotome-assisted precutting 25 , balloon-based strictures expansion 26 27 and calibrated wire-guided dilation techniques 28 mirror our findings regarding the critical importance of ductal preparation. Integration of enhanced visualization platforms, particularly SpyGlass-directed retrieval 29 , represents a significant advancement that complements conventional fluoroscopy, enabling direct visualization in tortuous ducts—a challenge we frequently encountered in cases with α- or S-shaped configurations. Furthermore, self-made modified devices such as Zimmer et al.’s 8-wire basket 30 , Ng et al.’s guidewire-assisted microsnare 31 and Kurihara et al.’s guidewire-assisted “zipline” technique 32 offer promising alternatives for narrow duct anatomy, expanding the armamentarium available for complex retrieval scenarios. These technical developments collectively represent an evolving paradigm that enhances success rates for endoscopic management for proximally migrated stents ( Table 5 ).

**Table:** **Table 5** Novel pancreatic stent retrieval methods over the past 5 years.

**Core point**	**Method**	**Instruments**	**Operative process**	**Reference**
Channel establishment	A basket catheter for peroral cholangioscopy through a biliary plastic stent pusher tube	8.5F plastic biliary stent pusher, basket catheter	Insert the biliary stent pusher tube anterior to the migrated pancreatic stent. Grasp the stent using the basket catheter via the pusher tube. Retract the stent into the pusher tube for successful removal.	23
	The “zipline” technique	Guidewire-assisted biopsy forceps with nylon thread	Place a guidewire. Advance the biopsy forceps along the guidewire using the nylon thread. Extract the pancreatic stent.	32
Duct dilation	A novel device delivery system	A novel device delivery system consisting of a slim-tip guiding catheter and a push tube, 1-mm biopsy forceps	Position the guidewire at the proximal end of the pancreatic duct. Insert the delivery system near the migrated stent. Deploy the biopsy forceps via the sheath to grasp the stent. Retract the forceps into the sheath for retrieval.	24
	The guidewire orientation of dental floss and the foreign body forceps	Cystotome, dental floss-guided retriever, foreign body forceps	Incise the strictured pancreatic duct using a cystotome. Place a guidewire. Extract the pancreatic stent using foreign body forceps guided by dental floss.	25
	Balloon catheter dilation	0.025-inch guidewire, 4 mm balloon catheter, basket catheter	Place a guidewire. Dilate the anastomosis using the balloon catheter. Grasp and remove the migrated stent with the basket catheter.	26
	Balloon catheter dilation	10 mm balloon catheter, rat-tooth forceps	Perform balloon catheter dilation. Grasp the distal end of the stent with rat-tooth forceps and extract.	27
Visualization	Using a new mini-basket and pushing catheter	7F contrast delivery catheter, spyglass retrieval basket	Measure the distance from the papilla to the stent tip. Position the contrast catheter at the stent tip under guidewire guidance. Deploy the SpyGlass basket to capture and retrieve the stent.	28
	SpyGlass DS system	SpyGlass DS system, SpyBite forceps	Perform a 5-mm pancreatic sphincterotomy (papillary orifice dilation). Visualize the pancreatic duct using the SpyGlass DS system. Identify and retrieve the stent using SpyBite forceps through the SpyGlass DS system.	29

**Fig. 2:**
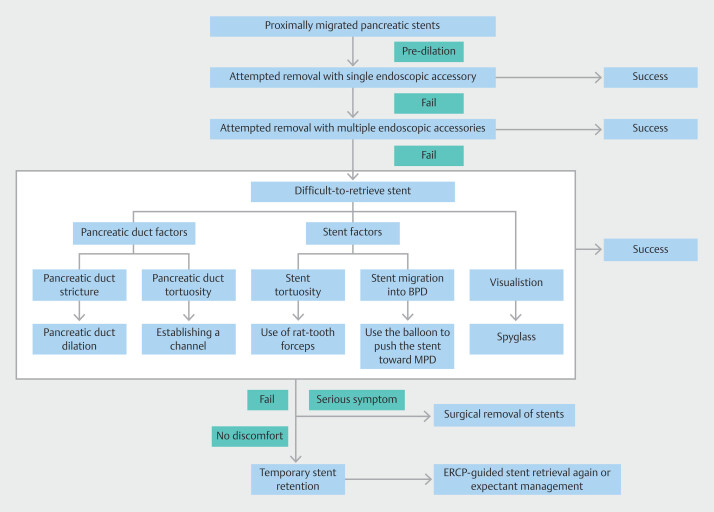
Empirical strategy for endoscopic removal of proximally migrated pancreatic stent.

Based on findings from this study and our institutional experience, we developed an endoscopic intervention strategy for migrated stents, achieving clinically satisfactory outcomes ( Fig. 2 ). This algorithm incorporates the concept of the difficult-to-retrieve stent as a critical clinical decision point, guiding endoscopists to transition from conventional techniques to advanced retrieval methods, thereby optimizing outcomes while mitigating procedure risks.

However, this study has several inherent limitations that warrant consideration. First, the relatively small sample size may compromise statistical power and limit the generalizability of the findings. Second, the retrospective design introduces potential selection bias and restricts rigorous control of confounding variables, particularly given the incomplete systematic collection of certain clinical parameters (e.g., degree of ductal stricture). Furthermore, the proposed difficult-to-retrieve stent concept, although clinically useful, requires external validation in future studies. Its predictive accuracy could be enhanced by incorporating imaging characteristics such as stricture severity and migration depth to develop a preoperative risk-stratification model. Despite these constraints, to our knowledge, this investigation represents one of the largest cohort analyses to date focusing on proximally migrated pancreatic stents. By analyzing case data from a large volume endoscopic center with a comprehensive literature review of advanced retrieval techniques, we have preliminarily characterized clinical characteristics of endoscopic retrieval for migrated pancreatic stents and developed evidence-based algorithmic recommendations for endoscopic management.

## Conclusions

In summary, these findings provide valuable clinical insights to guide endoscopic retrieval of migrated stents and future prospective studies with standardized data collection protocols are needed to validate our proposed management paradigm.
